# Tofacitinib to prevent anti-drug antibody formation against LMB-100 immunotoxin in patients with advanced mesothelin-expressing cancers

**DOI:** 10.3389/fonc.2024.1386190

**Published:** 2024-04-19

**Authors:** Nebojsa Skorupan, Cody J. Peer, Xianyu Zhang, Hyoyoung Choo-Wosoba, Mehwish I. Ahmad, Min-Jung Lee, Shraddha Rastogi, Nahoko Sato, Yunkai Yu, Guillaume Joe Pegna, Seth M. Steinberg, Shelley S. Kalsi, Liang Cao, William D. Figg, Jane B. Trepel, Ira Pastan, David FitzGerald, Christine Alewine

**Affiliations:** ^1^Laboratory of Molecular Biology, Center for Cancer Research, National Cancer Institute, National Institutes of Health, Bethesda, MD, United States; ^2^Clinical Pharmacology Program, Center for Cancer Research, National Cancer Institute, National Institutes of Health, Bethesda, MD, United States; ^3^Biostatistics and Data Management Section, Center for Cancer Research, National Cancer Institute, National Institutes of Health, Bethesda, MD, United States; ^4^Office of Research Nursing, Center for Cancer Research, National Cancer Institute, National Institutes of Health, Bethesda, MD, United States; ^5^Developmental Therapeutics Branch, Center for Cancer Research, National Cancer Institute, National Institutes of Health, Bethesda, MD, United States; ^6^Genetics Branch, Center for Cancer Research, National Cancer Institute, National Institutes of Health, Bethesda, MD, United States; ^7^Medical Oncology Program, Center for Cancer Research, National Cancer Institute, National Institutes of Health, Bethesda, MD, United States; ^8^Hematology Consult and Graduate Medical Section, National Heart Lung and Blood Institute, National Institutes of Health, Bethesda, MD, United States

**Keywords:** JAK inhibition, immunotoxin, anti-drug antibodies, pericarditis, mesothelin

## Abstract

**Background:**

LMB-100 is a mesothelin (MSLN)-targeting recombinant immunotoxin (iTox) carrying a Pseudomonas exotoxin A payload that has shown promise against solid tumors, however, efficacy is limited by the development of neutralizing anti-drug antibodies (ADAs). Tofacitinib is an oral Janus Kinase (JAK) inhibitor that prevented ADA formation against iTox in preclinical studies.

**Methods:**

A phase 1 trial testing LMB-100 and tofacitinib in patients with MSLN-expressing cancers (pancreatic adenocarcinoma, n=13; cholangiocarcinoma, n=1; appendiceal carcinoma, n=1; cystadenocarcinoma, n=1) was performed to assess safety and to determine if tofacitinib impacted ADA formation. Participants were treated for up to 3 cycles with LMB-100 as a 30-minute infusion on days 4, 6, and 8 at two dose levels (100 and 140 µg/kg) while oral tofacitinib was administered for the first 10 days of the cycle (10 mg BID). Peripheral blood was collected for analysis of ADA levels, serum cytokines and circulating immune subsets.

**Results:**

The study was closed early due to occurrence of drug-induced pericarditis in 2 patients. Pericarditis with the combination was not reproducible in a transgenic murine model containing human MSLN. Two of 4 patients receiving all 3 cycles of treatment maintained effective LMB-100 levels, an unusual occurrence. Sustained increases in systemic IL-10 and TNF-α were seen, a phenomenon not observed in prior LMB-100 studies. A decrease in activated T cell subsets and an increase in circulating immunosuppressive myeloid populations occurred. No radiologic decreases in tumor volume were observed.

**Discussion:**

Further testing of tofacitinib to prevent ADA formation is recommended in applicable non-malignant disease settings.

**Clinical trial registration:**

https://www.clinicaltrials.gov/study/NCT04034238.

## Introduction

Pancreatic cancer continues to have a very poor prognosis, with a 5-year overall survival rate of only 13% (1). Even though pancreas cancer represents approximately 3% of new cancer cases, it is the third leading cause of cancer death, and is projected to become the 2^nd^ within the next decade (1). Pancreatic ductal adenocarcinoma (PDAC) is the most common pancreatic cancer histology, accounting for >85% of pancreatic cancer cases (2). Most PDAC patients are diagnosed at incurable advanced stages. Combination chemotherapy was established as the standard of care for fit patients with locally advanced and metastatic PDAC a decade ago (3, 4). Since then, novel targeted modalities that have revolutionized care for other cancer types have only demonstrated activity in a minority of PDAC patients. Anti-CTLA4 and anti-PD(L)1 immune checkpoint inhibitors benefit ~2% of PDAC patients with mismatch repair deficient tumors but have no appreciable response rate in others (5, 6). Five to 10% of PDAC tumors have mutations in DNA-damage response genes (7, 8), a genotype that conveys exquisite sensitivity and response to platinum agents (9) and offers the possibility of benefit from PARP inhibitor therapy in the maintenance setting (10). The development of effective targeted therapies for other PDAC patients has been elusive. KRAS mutation is the primary oncogenic driver in PDAC and occurs in more than 90% of patients. While KRAS was long considered an “undruggable” target (11), recent breakthroughs have led to new anti-KRAS agents. Sotorasib is a KRAS inhibitor specific for the G12C mutation and has demonstrated efficacy in the ~3% of PDAC patients who harbor this mutation (12). Inhibitors of other KRAS mutations that are more common in PDAC (such as G12D) and even pan-RAS inhibitors are currently being investigated in early stage clinical trials (13). More efficacious and less toxic therapies for PDAC are desperately needed.

Mesothelin (MSLN) is a cell surface glycoprotein with expression typically limited to the mesothelial surfaces of the pleura, peritoneum, and pericardium. In addition, many solid tumors, especially mesothelioma, epithelial ovarian cancer, and PDAC, highly express MSLN, making MSLN a popular target for antibody-based therapeutics (14). LMB-100 is a second-generation MSLN-targeted immunotoxin (iTox) designed to bind and kill cells that express MSLN. LMB-100 contains a MSLN-binding Fab bearing a Pseudomonas exotoxin A (PE) payload. When the anti-MSLN Fab binds to MSLN-expressing cells the iTox is internalized through endocytosis and PE translocates to the cytosol. PE is an enzyme that irreversibly modifies elongation factor-2 via ADP-ribosylation, preventing new protein synthesis. This induces apoptosis in susceptible cell types (15).

LMB-100 has been tested in several clinical trials where the maximum tolerated dose (MTD) was established for the single agent, and in combination with the PDAC standard chemotherapy drug nanoalbumin-bound (nab)-paclitaxel (16–18). The most common toxicity was capillary leak syndrome (CLS), as seen previously with other iTox. Although LMB-100 was rationally designed to be less immunogenic than its predecessor iTox (19, 20), neutralizing anti-drug antibodies (ADAs) developed in all patients following serial administration of LMB-100. ADA development almost universally occurred within 2 cycles of treatment, resulted in zero or near-zero plasma peak concentration (C_max_), and was also associated with infusion-related reaction.

ADA development against iTox bacterial toxin payloads is not a new problem in the field and has been particularly problematic for drugs designed to treat solid tumor patients (21). Trials co-administering iTox with rituximab, cyclophosphamide or cyclosporin A all failed to reduce ADA formation (22–24) By depleting both T and B cells, use of a pentostatin/cyclophosphamide regimen successfully delayed ADA formation but this came at the cost of significantly increased toxicity and prolonged lymphocyte depletion (25). Recently, Onda and colleagues demonstrated that co-administration of the JAK (Janus Kinase) inhibitor tofacitininb delayed ADA formation in mice serially inoculated with iTox through multiple treatment cycles (26). Tofacitinib is an oral, well-tolerated, reversible inhibitor of JAK-STAT lymphocyte signaling (27), and is approved by the FDA for chronic treatment of multiple autoimmune diseases including ulcerative colitis (28), and rheumatoid arthritis (29). Tofacitinib inhibits both JAK3 and JAK1 signaling by preventing recruitment of STAT transcriptional factors that are critical for the production of multiple interleukins (IL) that regulate lymphocyte activation and differentiation, such as IL-4, IL-6 and IL-10 family members (30, 31). Administration of tofacitinib to mice resulted in decreased numbers of pro-B cells, decreased IgG1 and IgG2a production, and diminution of antigen-specific IgG1 responses, likely through suppression of IL-4 signaling, all of which likely contribute to ADA suppression (26). Separate pre-clinical studies examining the combination of tofacitinib with iTox additionally demonstrated that tofacitinib enhanced iTox delivery and anti-tumor efficacy (32).

Given the lack of serious acute toxicity caused by tofacitinib in patients, its ease of administration, its short half-life which allowed for rapid reversal of immune suppression (and return of lymphocyte-driven anti-tumor activity) upon drug hold, and potential to enhance iTox delivery, tofacitinib was chosen for advancement to clinical trial in combination with LMB-100 over other agents found to delay ADA development in pre-clinical studies (33). Here, we describe the results of a phase 1 trial examining the combination of LMB-100 iTox with tofacitinib in patients with advanced gastrointestinal (GI) malignancies including PDAC.

## Methods

### Study approval

The study protocol was approved by the NCI Institutional Review Board and written informed consent was obtained from all participants. All animal experiments were performed in accordance with NIH guidelines and approved by the NCI Animal Care and Use Committee.

### Study design and treatment

This open-label, phase I study was conducted at the NCI Center for Cancer Research (Bethesda, MD; NCT04034238). The study was conducted in accordance with FDA regulations and Good Clinical Practice guidelines. The study design is outlined in **Supplementary Figure 1A** and consisted of a dose escalation and a dose expansion cohort. The primary objective for the dose escalation cohort was to establish a maximum tolerated dose (MTD) for use in the Phase I dose expansion. All participants received tofacitinib (10mg PO daily) for days 1-10 in combination with LMB-100 (30-minute infusion, dose as per escalation schema) on days 4, 6, and 8 of each 21-day cycle for a maximum of 3 cycles. Pre-medication with diphenhydramine, acetaminophen and histamine-2 blocker (such as ranitidine) was administered to all participants prior to LMB-100 infusion. Due to the black box warning that tofacitinib could increase thrombotic risk, in addition to the baseline thrombotic risk related to pancreatic cancer, all patients except Patient 01 also received at least prophylactic dosing of an appropriate anticoagulant throughout study treatment if not already on treatment doses for a prior thrombotic event. A 3 + 3 design utilizing 2 dose levels was used to establish safe LMB-100 dose for this combination. The Phase I dose expansion was planned to include 15 patients, including those in the dose escalation cohort who met dose expansion arm eligibility criteria. The primary objective was to determine whether co-administration of tofacitinib delayed the formation of neutralizing anti-LMB-100 ADA’s through Cycle 2 of treatment as measured by peak plasma LMB-100 level (C_max_) following infusion. Prior published studies showed that 62% of patients receiving LMB-100 with or without nab-paclitaxel achieved Cycle 2 drug LMB-100 level higher than 100 ng/mL (16, 17), however, our re-analysis of these primary data suggested that LMB-100 Cycle 2 C_max_ >600 ng/mL better predicted lack of significant ADA formation than a 100 ng/mL threshold. Therefore, for this study, a threshold LMB-100 C_max_ of > 600 ng/mL during Cycle 2 LMB-100 administration was defined as indicative of no neutralizing ADA formation. Based on the same prior studies of LMB-100, 50% of patients were expected to achieve drug levels above threshold during Cycle 2. The current study was powered to detect an improvement to 80% from 50% and would be considered positive if 11 or more participants out of 15 met threshold drug level during cycle 2. Based upon summing standard binomial probabilities, the probability of obtaining 11 or more of 15 who would achieve drug levels above threshold during cycle 2 would be just 5.9% if the true probability for an individual was 50%, while the probability of this occurring would be 83.6% if the true probability for an individual was 80%. Other endpoints included a similar assessment of peak drug levels during Cycle 3, and description of the pharmacokinetics of LMB-100 in this combination.

### Patients

Eligible participants were ≥ 18 years old with histological confirmation of PDAC, extrahepatic cholangiocarcinoma (ECC), epithelioid subtype of mesothelioma or a solid tumor with ≥ 20% of tumor cells expressing mesothelin as measured by validated IHC assay. For the dose expansion arm, only patients with PDAC or extrahepatic cholangiocarcinoma were eligible. Participants in all cohorts must have received at least one prior standard systemic treatment for advanced disease. Other requirements included: evaluable disease (measurable per RECIST version 1.1 or by an appropriate tumor marker, e.g. CA 19-9), Eastern Oncology Cooperative Group performance status (ECOG PS) 0-2, adequate organ function, including left ventricular ejection fraction ≥ 50% and ambulatory oxygen saturation of > 88% on room air. See full inclusion and exclusion criteria in **Supplementary Methods**.

### Sex as a biological variable

Our clinical study examined male and female participants, and similar findings are reported for both sexes. Mouse co-clinical trial was performed in animals of both sexes.

### Clinical assessments

Adverse events (AEs) were graded using Common terminology Criteria for Adverse Events (CTCAE) version 5.0. Dose limiting toxicity (DLT) was defined as an occurrence of certain serious hematological toxicities or grade ≥ 3 nonhematological toxicity (clinically insignificant electrolyte abnormalities excluded) within 21 days of treatment initiation. For disease evaluable by imaging, objective response was assessed (using RECIST version 1.1) at baseline and 6 weeks after treatment initiation in participants who had received at least 1 cycle of therapy. For non-target disease response, serial tumor marker measurement was used.

### Pharmacokinetic, ADA, and serum cytokine measurements

**Figure 1:**
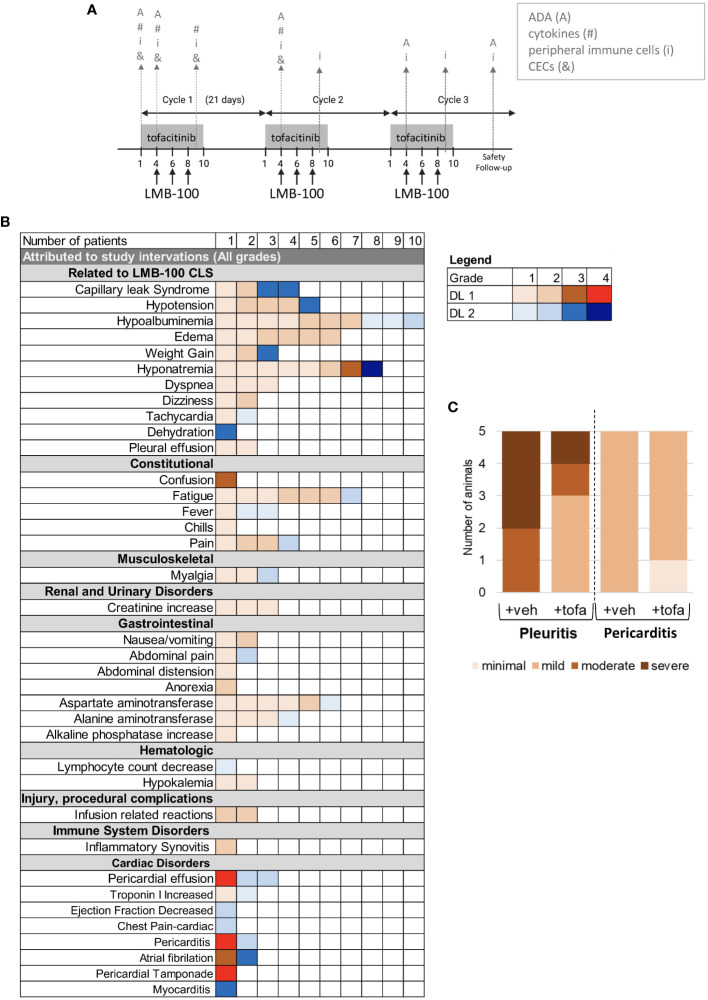
Study design and Adverse Events. **(A)** Tofacitinib and LMB-100 administration schedule. Gray arrows indicate sampling times for immune correlative studies. **(B)** Summary of adverse events attributed to study intervention. **(C)** Histogram delineating severity of serositis as graded on H&E by a trained pathologist in human mesothelin-expressing transgenic mice treated with LMB-100.

Timepoints for all correlative blood draws are shown in **Figure 1A**. Patient plasma LMB-100 concentrations and ADA levels were measured by validated ELISA through contract with Frederick National Laboratory for Cancer Research operated by Leidos Biomedical Research, Inc. as described previously (17). C_max_ values were recorded as observed values, while other parameters, namely AUC, half-life, clearance and volume of distribution, were calculated using noncompartmental methods and summarized arithmetically. Blood for serum cytokine evaluation was separated within 4 hours and stored in aliquots at -80°C until use. The samples were tested using clinically validated custom V-PLEX assay plates on an electrochemiluminescence platform, according to the manufacturer’s instructions (Meso Scale Discovery). The following cytokines were assessed: IL-17a, IFN-ɣ, IL-1β, IL-2, IL-4, IL-6, IL-8, IL-10, IL-12p70, IL-13, and TNF-α. For patients with cytokine levels below the limit of detection (LOD), an imputation method was used (i.e. LOD/√2) (34) to calculate the fold change.

### Immune subset and circulating endothelial cell analyses

Standard clinical lymphocyte phenotyping was performed by CLIA-certified clinical lab at NCI Clinical Center. For more intensive immuneanalysis, peripheral blood samples were collected in Cell Preparation Tubes™ with sodium citrate (BD Vacutainer CPT Tubes, BD Biosciences, San Jose, CA). Peripheral blood mononuclear cells (PBMCs) were obtained by centrifugation and viably frozen in liquid nitrogen until analysis. Immune subsets and functional markers were analyzed using multiparameter flow cytometry as described previously (18) and in **Supplementary Methods**. CEC analyses were performed as described previously (19).

### Murine studies of serositis

Msl transgenic mice, which have knock-in of human mesothelin ortholog into the murine mesothelin locus such that human mesothelin is expressed in the native distribution, were engineered and bred as described in (35). Mice (*Msln^ki/+^*) were treated with tofacitinib (25 mg/kg, 200 µL volume, twice daily by OG, days 1-6) plus LMB-100 (3.5 mg/kg IV, 200 µL volume, Days 4 and 6), oral vehicle (200 µL OG, days 1-6) plus LMB-100, or oral vehicle plus IV PBS (200 µL volume IV, Days 4 and 6). Tofacitinib citrate was purchased from SelleckChem (product S5001), dissolved in DMSO to 250 mg/mL for storage, then diluted into 0.5% methylcellulose, followed by 0.05% Tween20 and PBS just before use. LMB-100 iTox was manufactured by Roche and provided for these studies through a Collaborative Research and Development Agreement. Animals were euthanized 24 hours after last treatment. Tissue specimens were fixed in 10% neutral buffered formalin solution for 48-72 h at RT (HT501128, Sigma-Aldrich, USA), then sent to Molecular Histotechnology Laboratory (MHL) Core facility for all histologic studies. Analysis and quantification were performed by a trained veterinary pathologist affiliated with the MHL Core using criteria described in **Supplementary Methods**.

### Statistical analysis

Graphs were generated using Microsoft Excel, R Statistical Software (v4.1.1; R Core Team, 2021), BioRender or GraphPad Prism (Version 9.5.0) and statistical analyses were performed in Prism or R Statistical Software (v4.1.1; R Core Team, 2021).

### Data availability

Data are available upon reasonable request. Values for all data points in graphs are reported in the Supporting Data Values file.

## Results

### Patient characteristics

Sixteen patients were treated with LMB-100 infusion combined with oral tofacitinib between August, 2019 and November, 2020 (**Supplementary Figure 1A**). Thirteen patients were treated with 100 mcg/kg of LMB-100 (dose level [DL] 1), and 3 patients at 140 mcg/kg of LMB-100 (DL2). Four patients received 3 cycles, 3 patients received 2 cycles, and 8 patients received only 1 cycle of the study treatment. One patient started tofacitinib but never received LMB-100 due interval development of hyperbilirubinemia. One patient withdrew during the first cycle after developing deep venous thrombosis. Patient characteristics are summarized in **Table 1**. Most patients enrolled had PDAC. Notably, 87.5% of participants had liver metastases, 62.5% had lung metastases, and 12.5% had clinically apparent ascites upon enrollment.

**Table 1:** Baseline patient demographics and clinical characteristics.

Characteristics	Value
*No. of patients*	16
DL1 - no. (%)	13 (81.3)
DL2 - no. (%)	3 (18.8)
Age	
Median (range)	62 (31-85)
>70yr - no. (%)	6 (37.5)
*Male/Female - no. (%)*	11 (68.8)/5 (31.2)
WHO performance status - no./total no. (%)	
0	8
1	8
Tumor type- no./total no. (%)	
Pancreatic Ductal Adenocarcinoma (PDAC)	13 (81.3)
Intrahepatic Cholangiocarcinoma	1 (6.3)
Cystadenocarcinoma	1 (6.3)
Appendiceal carcinoma	1 (6.3)
Sites of metastatic disease for all - no. (%)	
Liver	14 (87.5)
Lung	10 (62.5)
Ascites	2 (12.5)
Other	12 (75)
Prior lines of therapy for all	
Median (range)	3 (1-5)
Surgical resection - no. (%)	5 (31.3)
Radiation	9 (56.3)
*Baseline CA 19-9 for PDAC - median (range)*	1937.5 (0.8-251,100.0)

DL, dose level.

### Safety and tolerability

All participants received LMB-100 with tofacitinib per the schedule shown in **Figure 1A**. Adverse Events (AEs) attributed to the study intervention are tabulated in **Figure 1B** and serious toxicities considered unrelated to the study intervention are reported in **Supplementary Figure 1B**. The most common AEs attributed to study treatment were the CLS-related toxicities of hypoalbuminemia (62%), hyponatremia (50%), edema (37%) and fatigue (37%). Despite this only 2 participants developed significant CLS, previously defined as >5kg weight gain from edema. One patient was treated on DL2 and one on DL1. AST elevation (37%) unrelated to CLS was also frequently seen and attributed to study drug. DL1 was established as the MTD for the LMB-100/tofacitinib combination after 2 of 3 patients enrolled on DL2 experienced dose-limiting toxicity (DLT) of grade 3 CLS. Pt09 experienced hyponatremia (grade 4), and atrial fibrillation (grade 3), dehydration, hypotension, and weight gain as part of CLS from LMB-100. Pt09 was able to receive cycle 2 at a reduced LMB-100 dose (100 mcg/kg) without further AEs. In Pt04, severe CLS symptoms were accompanied by myocarditis (grade 3) and serosal membrane toxicity (pericarditis, grade 2). Due to the weight-based dosing scheme, this 115 kg patient received the highest dose of LMB-100 in the study, resulting in high peak LMB-100 plasma concentration (C_max_ = 3082 ng/mL, **Supplementary Figure 1C**). The protocol was subsequently amended to cap maximum LMB-100 dose to what would be given for a 100 kg person. All other participants were treated at DL1 from the outset. Pt19, the last patient treated, developed treatment-related pericardial effusion with tamponade (grade 4), pericarditis (grade 4), and atrial fibrillation (grade 3), after the C1D6 dose of LMB-100. The occurrence of serious cardiac toxicity due to serosal membrane irritation in a second participant prompted the study team to close enrollment early for safety.

While pericarditis and other serosal membrane toxicities are theoretical on-target off-tumor toxicities of mesothelin-targeted therapeutics like LMB-100, these were the first two occurrences of pericarditis in pancreatobiliary cancer patients receiving LMB-100. Since peritoneal/cardiac biopsy of study patients was not feasible, we evaluated the combination for increased serosal toxicity in an informative transgenic rodent model. Specifically, Msl transgenic mice that have knock-in of full-length human *MSLN* into the murine *Msln* locus were treated with LMB-100 ± tofacitinib and histologic evidence of serositis in the pleura and pericardium was graded by a veterinary pathologist blinded to the treatment groups. Robust expression of human MSLN in the pleura and pericardium of these mice has previously been demonstrated (35). No serosal inflammation was identified in the control mice treated with vehicle (data not shown). Similar amounts of serosal inflammation were seen in LMB-100-treated mice regardless of whether they also received tofacitinib (**Figure 1C**). Pericardial inflammation was less than pleural inflammation and no mice developed pericardial effusion or tamponade. Like 13 of the 15 patients treated with LMB-100 on the clinical study, the mice had no clinical signs or symptoms of serosal membrane toxicity even when co-treated with tofacitinib and could not be used as a model to further investigate the etiology of this unexpectedly severe toxicity. It was not feasible to determine whether the transgenic mice had lower levels of pericardial MSLN expression than the patients who developed cardiac toxicity, one possible explanation for this discrepancy.

Only 2 patients experienced significant LMB-100-induced CLS on this study, as defined by >5 kg weight gain from CLS-related edema. Previously we have shown that the percentages of apoptotic circulating endothelial cells (CECs) increases in proportion to severity of LMB-100-induced CLS while the numbers of viable CECs remained unchanged (17). Here, we saw a trend towards transient increases in apoptotic and viable CECs following LMB-100 + tofacitinib treatment (C1D9) as compared to baseline (C1D1) (**Supplementary Figures 1D, E**). Notably, Pt09, who developed CLS (grade 3) did not have follow-up samples available and could not be included in the analysis.

### Pharmacokinetic analysis and relationship to ADA development

**Figure 2:**
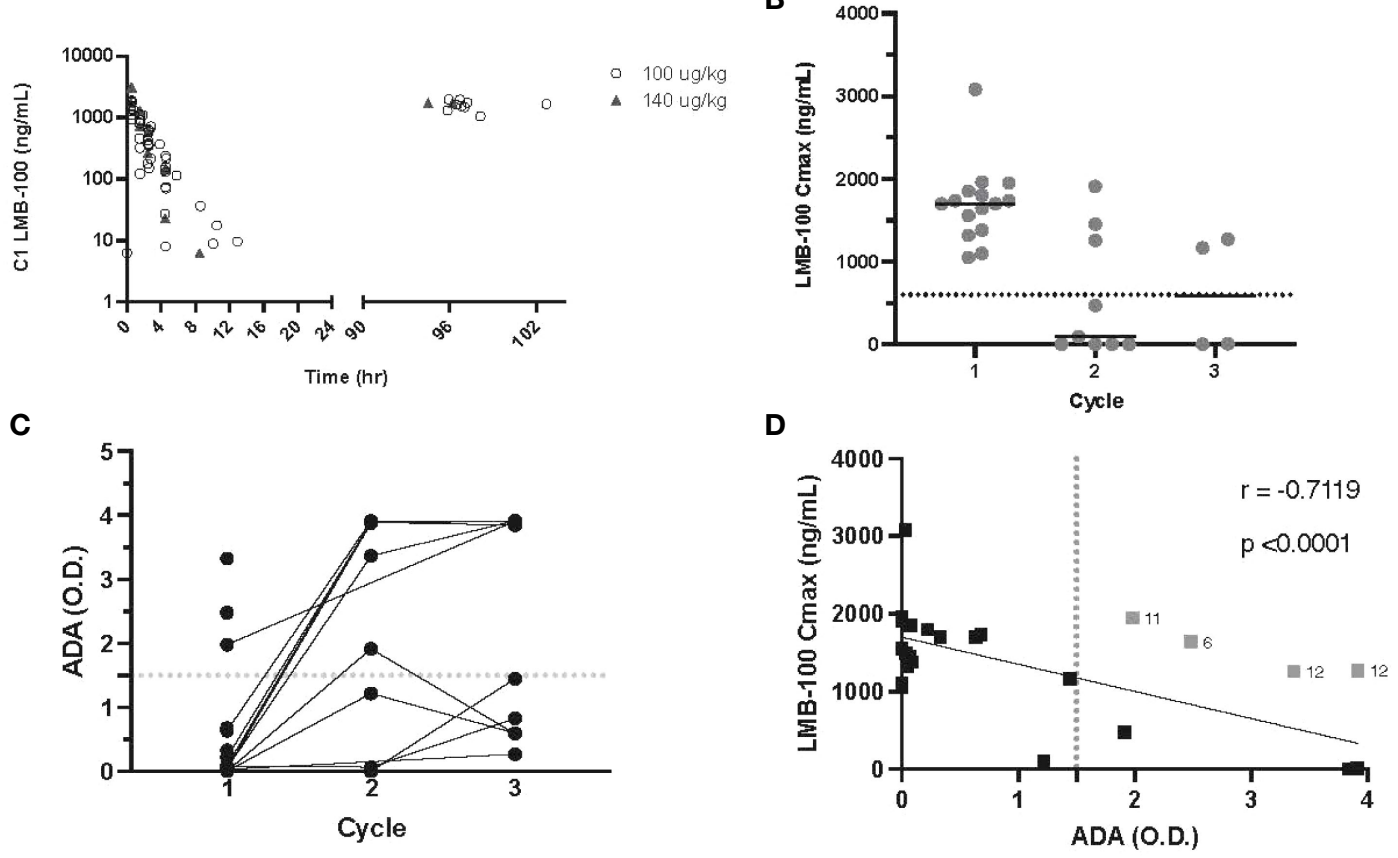
LMB-100 pharmacokinetics and ADA formation. **(A)** LMB-100 plasma concentration for each patient during C1. **(B)** LMB-100 C_max_ by patient and cycle. The solid line indicates the median. The dashed line indicates LMB-100 previously established threshold of efficacy (600 ng/mL). **(C)** ADA levels for individual patients before each indicated cycle of treatment. **(D)** Plot of LMB-100 C_max_ versus ADA for each patient. Solid line shows fit for simple linear regression. The vertical dotted line indicates 1.5 OD cut-off associated with low LMB-100 C_max_ in previous clinical studies. Patients with LMB-100 C_max_ > threshold despite ADA > cut-off are indicated in gray with neighboring numeral defining patient #. Pt12 was an outlier for both C2 and C3 treatment.

Serial LMB-100 plasma drug concentrations were available for 15 participants. **Supplementary Table 1** provides a summary of all pharmacokinetic parameters. LMB-100 concentration declined in a mono-exponential manner following infusion irrespective of the dose level as seen previously (**Figure 2A**). Numerically, the half-life, clearance and volume of distribution were also similar to previous studies of single-agent LMB-100 and LMB-100 given with chemo- or immunotherapy (16, 17). Tofacitinib co-administration did not affect first-dose pharmacokinetic parameters of LMB-100.

As seen in prior studies of LMB-100, peak LMB-100 plasma concentration (C_max_) trended down with repeated iTox administration. However, 2 out of 4 patients who received cycle 3 continued to have plasma drug levels higher than the 600ng/mL pre-defined study threshold for “good” drug level (**Figure 2B**). Concordant with decreasing peak blood levels of LMB-100, the ADA titer rose in most patients prior to Cycle 2 of treatment (**Figure 2C**). In prior clinical studies of LMB-100, LMB-100 C_max_ and ADA titers were inversely correlated, and ADAs >1.5 O.D. were predictive of undetectable LMB-100 (18). The inverse correlation persists with co-administration of tofacitinib (p -0.7119), but several patients had peak LMB-100 levels higher than the effective threshold despite ADA >1.5 O.D. (**Figure 2D**). These data suggest that tofacitinib co-administration may delay the development of neutralizing ADAs or reduce their effect on peak drug levels in some patients.

### Anti-tumor activity of LMB-100 + tofacitinib combination

There were no RECIST-defined radiologic responses amongst the 14 patients who received at least 1 dose of LMB-100. Of the 9 patients evaluable for serum tumor marker assessment, 2 participants had PDAC tumor marker CA 19-9 decrease by >50% from baseline (**Supplementary Figure 2**), but the decline was most likely due to factors outside of the intervention (ie. recent biliary stenting) rather than a true treatment response. Clinically significant anti-tumor activity was not observed with the LMB-100/tofacitinib combination.

### Effect of treatment on plasma cytokines

**Figure 3:**
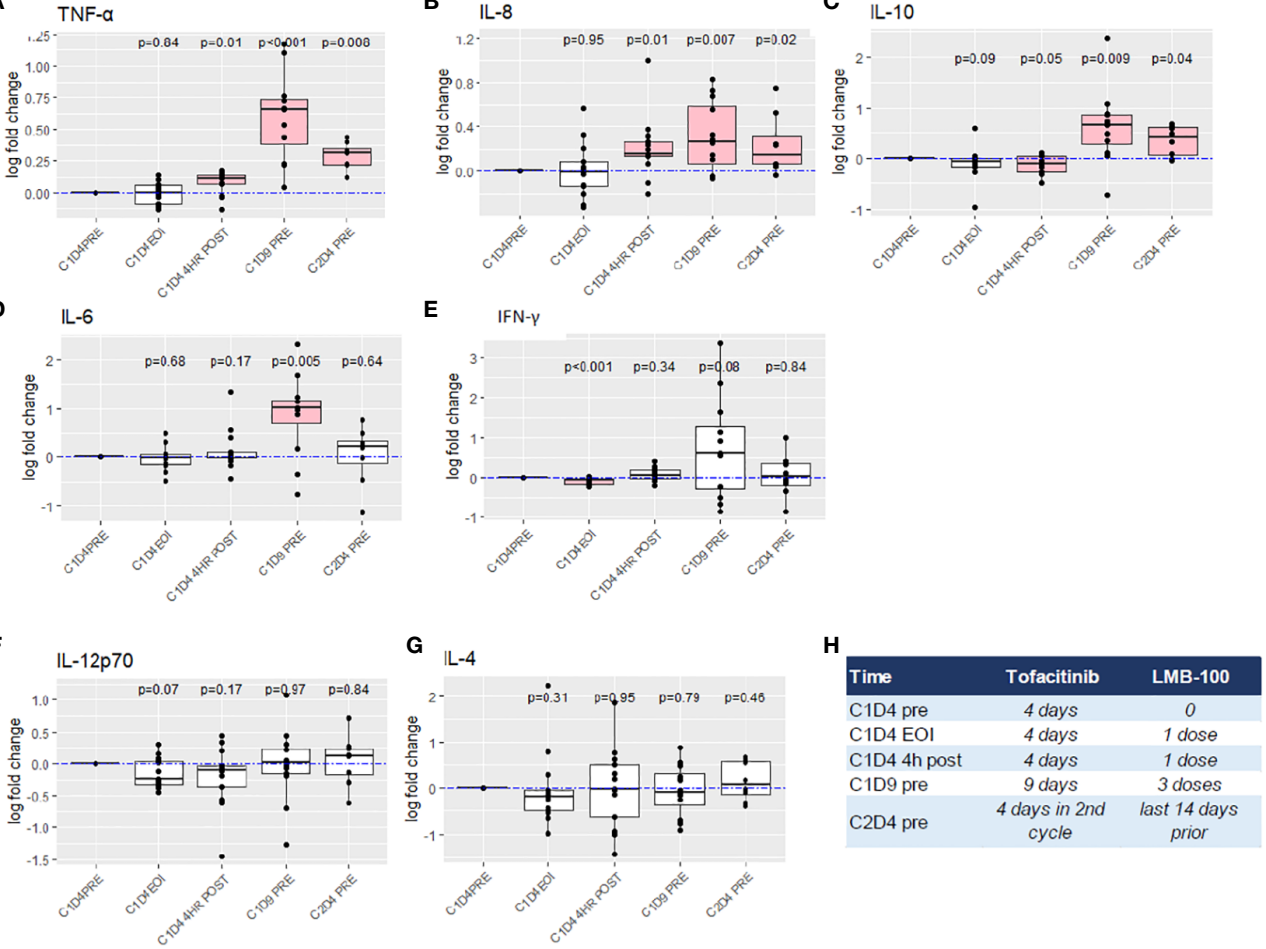
Fold change analysis of peripheral blood cytokine levels. **(A–G)** Boxplot summary with exact p-values by Wilcoxon signed-rank test are reported for indicated cytokines. The dashed blue lines indicate log fold change of 0 (no change). Horizontal black bars indicate the median for each group. Boxplot indicates the interquartile range. Outlier points are connected by vertical lines. Pink boxes indicate cohort measurements with *p <*0.05 as compared to C1D4 baseline. Imputation was required for 5 values below LOD for IL-4. **(H)** Reference chart defining amount of treatment received at time of each cytokine measurement.

Peripheral blood was assessed for the concentration of 11 different cytokines at the timepoints indicated in **Figure 1A**. Individual patient level data is plotted in **Supplementary Figure 3**. Thirteen patients had baseline (C1D4, status-post tofacitinib x3 days, but prior to LMB-100 infusion) values reported. Fold change in the concentration of 7 cytokines following LMB-100 administration are shown in **Figures 3A–H**. TNF-ɑ, and IL-8, concentrations increased, while IL-10 concentrations decreased within 4 hours of LMB-100 administration. Subsequently, all 3 cytokines persisted at concentrations higher than baseline through the start of Cycle 2 (**Figures 3A–C**). IL-6 concentration had spiked by the time all 3 doses of LMB-100 were administered, but then returned to baseline before Cycle 2 LMB-100 administration (**Figure 3D**). A significant decrease in IFN-ɣ was observed at the end of the first LMB-100 infusion that resolved within 4 hrs (**Figure 3E**). By completion of all 3 LMB-100 infusions on C1D9, IFN-ɣ concentrations remained low in some patients but spiked in others. No significant changes in IL-4 and IL-12p70 concentrations were observed following LMB-100 administration (**Figures 3F, G**). The remaining 4 cytokines assayed had concentrations largely below the limit of detection (**Supplementary Table 2**) and could not be used to make appropriate statistical inference (**Supplementary Table 3**).

### Effect of treatment on peripheral immune cell subsets

**Figure 4:**
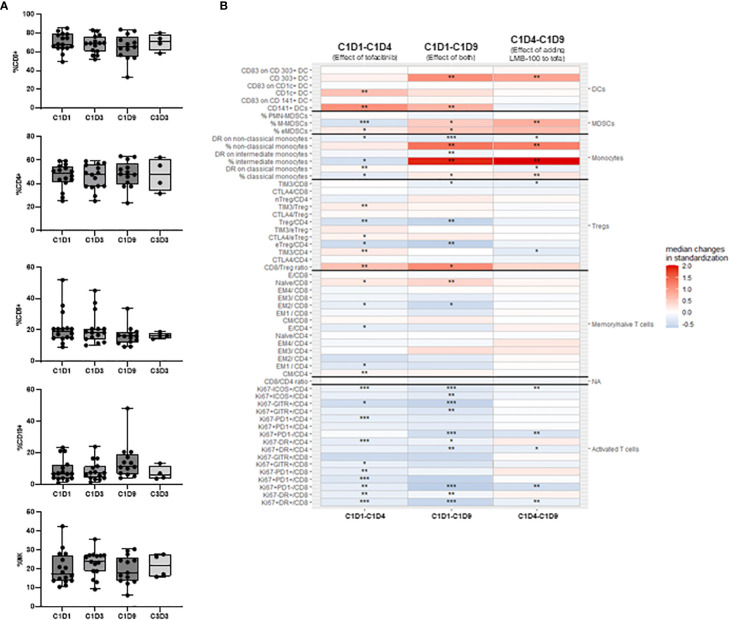
Peripheral blood immune subsets. **(A)** Peripheral blood lymphocyte percentages. No statistically significant changes were identified using Kruskall-Wallis non-parametric test adjusted with Dunn’s test for multiple comparisons. **(B)** Flow cytometric assessment of peripheral immune cells reporting intrapatient changes for all 58 cell types assessed. Each heatmap color block shows median changes in percentage of each cell subset for all evaluable patients over the indicated time period. C1D1 timepoint baseline occurred pre-tofacitinib and pre-LMB-100 administrations. Other timepoints are as defined in **Figure 3H**. Missing values were assumed to be random and were excluded from the analysis. To explore paired differences, an exact Wilcoxon signed rank test was performed for each cell type, to calculate p-value. The analysis was not adjusted for multiple comparisons. Levels of significance: * *p <*0.05, ** *p <*0.01, *** *p <*0.001.

Peripheral immune cell subsets were assessed at the indicated time points as shown in **Figure 1A**. Treatment had no effect on the percentages of T (total, CD4+ or CD8+), B or NK cell subsets (**Figure 4A**). Interestingly, intensive analysis of T cell populations by multi-parametric flow cytometry demonstrated that the ratio of CD8/Treg cells increased with initiation of tofacitinib (C1D1-C1D4) and even further with addition of LMB-100 (C1D4-C1D9). By contrast, most activated T cells subsets decreased through the course of LMB-100 treatment but were amplified above baseline before the start of LMB-100 during Cycle 2 (**Figure 4B**; **Supplementary Figure 4**). The percentage of naïve cells among total CD8+ T cells increased with initiation of tofacitinib (C1D1-C1D4), and further increased following LMB-100 treatment (C1D1-C1D9). More detailed subsets of B and NK cell populations were not assessed.

Monocyte, myeloid derived suppressor cells (MDSCs), and dendritic cell (DC) populations were significantly perturbed by treatment administration (**Figure 4B**). LMB-100 treatment increased the percentage of non-classical, intermediate, and classical monocytes, although HLA-DR expression on these cells largely declined (**Figure 4B**). However, the relative population of monocytes declined by the start of Cycle 2 LMB-100 treatment (**Supplementary Figure 4**). Populations of CD303+ DCs, monocytic MDSCs (M-MDSCs), and early MDSCs (eMDSCs) also had large increases following LMB-100 treatment (**Figure 4B**) which persisted through to the start of Cycle 2 (**Supplementary Figure 4**). CD1c+ and CD141+ DCs transiently increased following introduction of tofacitinib. Significant changes in CD83 expression, a functional marker of maturation, on DCs were not observed.

## Discussion

In our study, the combination of LMB-100 with tofacitinib was judged unsafe due to multiple events of pericarditis. This toxicity of the combination could not be reproduced in a transgenic murine model with serosal membrane expression of the MSLN antigen that LMB-100 targets, such that the mechanism for increased serosal membrane toxicity in the presence of JAK inhibitor remains unclear. Unfortunately, the combination also lacked significant anti-tumor efficacy. This led to our assessment of ADA formation being underpowered. Despite this, several patients maintained therapeutic LMB-100 levels throughout the planned treatment course of 3 cycles, suggesting tofacitinib may provide benefit in preventing the rapid drug clearance that typically occurs in the presence of neutralizing ADAs.

The primary objective of this study was to assess whether tofacitinib co-administration could delay ADA formation against LMB-100, which is a significant barrier to iTox efficacy in solid tumors. Unfortunately, due to the early closure for safety, and the inadequate anti-tumor efficacy of the combination, there were an insufficient number of evaluable patients receiving at least 2 cycles of treatment to definitively assess our ADA prevention endpoint. Numerically, only 3 of 9 patients (33%) achieved drug levels above threshold during the second cycle, when prior studies have suggested 50% should reach this milestone. With the early closure, the study lacked power to draw statistical conclusions about this ratio, however, the data revealed hints that tofacitinib might affect ADA drug neutralization. Notably, LMB-100 C_max_ remained at therapeutic levels in 2 of 4 patients who received all 3 planned cycles of therapy. Although these numbers are small, it is highly unusual for any patient receiving LMB-100 to have measurable peak drug levels by Cycle 3. Furthermore, multiple patients with high ADA titers continued to have LMB-100 C_max_ above the efficacy threshold, suggesting that our intervention in pre-treating with tofacitinib decoupled the inverse relationship between ADAs and peak drug levels in some patients.

While iTox remains experimental for the treatment of solid tumors, efficacy of many standard of care, life-saving protein drugs is limited by ADA development. These include enzyme replacement therapies (ERTs) for lysosomal storage diseases (LSD) and coagulation factor deficiencies (36). With LMB-100, development of ADAs has previously been linked with near zero peak plasma drug concentrations. For ERT, there is also strong evidence that ADAs develop with repeated administration (37–40). In some, such as Pompe’s disease, ADA formation to ERT is associated with devastating clinical outcomes (41, 42). Similarly, development of host IgG “inhibitors” against exogenous Factor VIII (hemophilia A) or Factor IX (hemophilia B) occurs in around 30% and 5% of hemophilia patients, respectively (43–45). The presence of these ADA “inhibitors” is associated with higher mortality risk in Hemophilia A (46), and anaphylaxis in Hemophilia B (47). Emicizumab, a novel antibody that mimics a key function of activated Factor VIII (48), provides one alternative for hemophilia patients with neutralizing antibodies against coagulation factors, but ADAs to emicizumab also occur (49–51). A further, definitive exploration of the ADA preventative function of tofacitinib in one of these populations may be warranted, as safety of ERT combined with tofacitinib is highly likely, and efficacy in stopping a highly aggressive cancer is not required for study participants to remain evaluable through sufficient cycles of treatment to make an assessment.

Pericarditis, a previously theoretical on-target off-tumor serosal membrane toxicity of MSLN-targeted therapeutics like LMB-100, was observed in two patients with pancreatobiliary cancer receiving the LMB-100 plus tofacitinib combination. Overall, pericardial toxicity has been very rare in patients receiving MSLN-targeted therapeutics in the absence tumor infiltration into the pericardium (as occurs in many mesothelioma patients). It remains unclear why pericardium is largely spared by anti-MSLN antibody-drug conjugates, CAR T cells, and previous generation MSLN-targeted iTox SS1P. This report documents the first occurrences of pericarditis occurring in patients with pancreatobiliary cancer receiving LMB-100. While the first patient who developed pericarditis did receive the study’s highest dosage of LMB-100 due to the weight-based dosing schema, peak LMB-100 concentration in the patient with the most serious pericardial toxicity was similar to other participants on the study. This suggests that combination of LMB-100 with tofacitinib specifically increases the risk of cardiac serosal membrane toxicity for some patients receiving this MSLN-targeted intervention. For obvious safety reasons, we could not obtain pericardial tissue from our patients on study to examine what had occurred at the cellular level, and, unfortunately, we were unable to successfully model combination-induced pericardial toxicity in mice. It currently remains a mystery why LMB-100 combined with a strong anti-inflammatory drug such as tofacitinib would cause increased pericardial inflammation, but inflammation-related serious adverse events including pericardial effusion (grade 4) were also seen when LMB-100 was combined with rapamycin nanoparticle SEL-110, another lymphocyte signaling modulator (NCT03436732, results reported on clinicaltrials.gov). When considering changes in cytokine levels, we observed sustained increases in systemic IL-10 and TNF-α following initiation of LMB-100, increases which were not seen in prior studies lacking co-administration of tofacitinib. High serum levels of IL-10 alongside TNF-α have previously been seen in patients with autoimmune myocarditis (52, 53), but links between elevated TNF-α and pericarditis or pericardial effusions have not been observed (54, 55). Here, increases in IL-10 and TNF-α occurred in almost all patients on study, and were not more intense for those with serosal membrane toxicity, so it is unlikely that these systemic cytokines are associated with an increased pericardial inflammatory response. We speculate that a more localized process is responsible for pericarditis events and await future research in informative model systems to delineate a molecular mechanism for the paradoxical increase in serositis we have observed upon addition of JAK inhibitor.

CLS is a frequent DLT of iTox therapies (16, 56), and was also the DLT in this study. Our previous work identified endothelial damage as the likely cause of CLS, since the severity of iTox-induced CLS correlated with levels of apoptotic CECs (17). Given the timescale of effect, it was concluded that the endothelial damage was most likely secondary to a more proximal immune cell release of IL-8 and IFNγ that is directly caused by iTox (18). In our present study, IL-8 levels largely increased following LMB-100 treatment, a previously established effect of exposure to *Pseudomonas* exotoxin A (57), but IFNγ changes were more variable. Tofacitinib has previously been shown to suppress IFNγ levels in animal models (30) and human subjects (58) and may also be doing so here.

Anti-tumor activity of LMB-100 in past clinical and pre-clinical studies has been associated with T cell activation (18, 59, 60). Here, activated T cell subsets declined with initiation of therapy, a not unexpected outcome given the known activity of tofacitinib. It was hoped that limiting the tofacitinib course to 10 days of the 21-day cycle would allow for resumption of active T cell signaling (accompanied by lymphocyte-driven anti-tumor responses) during the latter half of each treatment cycle. While activated T cell subsets did rebound above baseline levels before the start of Cycle 2 LMB-100 treatment, this did not generate a successful anti-tumor effect. Notably, it was accompanied by increases in circulating immunosuppressive myeloid populations. Although tumor biopsy to directly assess changes in the TME was planned for this study and would be valuable in understanding outcomes, the study was largely conducted during the COVID pandemic which adversely affected feasibility of elective procedures, and patient tumor tissue was not available for analysis. Nevertheless, our data do not support further pursuit of this combination in solid tumors.

In conclusion addition of tofacitinib to LMB-100 increased toxicity but failed to enhance anti-tumor efficacy in our primarily pancreatobiliary patient population. This unfavorable clinical profile resulted in early study closure and insufficient power to definitively assess the role of tofacitinib in delaying or preventing ADA formation in this population. We observed decoupling of the inverse relationship between high-titer ADA formation and low drug levels of LMB-100 in a small number of patients able to receive the planned 3 cycles of therapy. Reassessment of tofacitinib as a means of preventing or delaying ADA formation caused by repeated administration of antigenic protein drugs may be more tractable in the LDS and/or hemophilia patient populations that require ERT.

## Data availability statement

The original contributions presented in the study are included in the article/**Supplementary Material**, further inquiries can be directed to the corresponding author/s.

## Ethics statement

The studies involving humans were approved by NIH Institutional Review Board. The studies were conducted in accordance with the local legislation and institutional requirements. The participants provided their written informed consent to participate in this study. The animal study was approved by NIH IC Animal Care and Use Committee. The study was conducted in accordance with the local legislation and institutional requirements.

## Author contributions

NSk: Data curation, Writing – original draft, Writing – review & editing, Formal analysis, Visualization. CP: Data curation, Writing – review & editing, Conceptualization, Formal analysis. XZ: Data curation, Writing – review & editing, Formal analysis, Investigation. HC: Writing – review & editing, Data curation, Formal analysis. MA: Data curation, Writing – review & editing, Investigation. M-JL: Data curation, Writing – review & editing, Investigation. SR: Data curation, Writing – review & editing, Investigation. NSa: Data curation, Writing – review & editing, Investigation. YY: Data curation, Writing – review & editing, Investigation. GP: Writing – review & editing, Investigation. SS: Writing – review & editing, Conceptualization, Formal analysis. SK: Writing – review & editing, Investigation, Methodology. LC: Writing – review & editing, Investigation, Project administration. WF: Writing – review & editing, Funding acquisition, Methodology, Project administration. JT: Data curation, Writing – review & editing, Methodology, Project administration. IP: Conceptualization, Writing – review & editing. DF: Conceptualization, Writing – review & editing, Formal analysis. CA: Conceptualization, Data curation, Writing – original draft, Writing – review & editing, Funding acquisition, Investigation, Methodology, Project administration, Supervision, Visualization.
