# Supplementary material for: Tofacitinib to prevent anti-drug antibody formation against LMB-100 immunotoxin in patients with advanced mesothelin-expressing cancers
Source: Front Oncol. 2024 Apr 19;14:1386190. doi: 10.3389/fonc.2024.1386190 (PMC11066227; doi:10.3389/fonc.2024.1386190)
Supplement: Supplementary file 1 [file DataSheet_1.docx]

**Supplemental Methods**

*Eligibility criteria*

Inclusion Criteria:

1. Patients must have histologically confirmed solid tumor malignancy for which no curative therapy exists.
2. Pancreatic adenocarcinoma, extrahepatic cholangiocarcinoma or epithelioid subtype of mesothelioma, as determined by NCI Laboratory of Pathology, OR for all other tumor types, at least 20% of tumor cells must express mesothelin. Determination can be made using archival tumor tissue or fresh biopsy if archival tumor tissue is not available.
3. All patients must have evaluable disease (i.e. measurable per RECIST 1.1. or by following CA19-9 tumor marker). Patients in the expansion cohort must have measurable disease, per RECIST 1.1.
4. Patients must have received at least one prior standard systemic treatment regimen for advanced disease OR be ineligible to receive available standards due to co-morbidities, prior toxicity, lack of standard options for tumor type, or having received all standards available for prior treatment of early stage disease OR have refused first-line standard systemic treatment but have received prior anti-cancer treatments.
5. Patients with dMMR/MSI-H disease must have received at least one prior anti-PD1 therapy, be ineligible to receive this treatment due to concurrent medical conditions, or have refused this therapy.
6. ECOG performance status (PS) 0-2.
7. Age >18 years. Because no dosing or adverse event data are currently available on the use of LMB-100 alone or in combination with tofacitinib in persons with <18 years of age, children are excluded from this study.
8. Patients must be more than 14 days removed from most recent minor surgical procedure (such as biliary stenting), 28 days from most recent major surgical procedure and 14 days from radiation therapy, systemic treatments (such as chemotherapy), or experimental drug treatment. All acute toxicities from prior treatment must have resolved to grade 1 or less except alopecia, anemia, peripheral neuropathy, or endocrinopathies corrected by replacement therapy.
9. Adequate hematological function: neutrophil count of ≥ 1.5 x 103 cells/μL, platelet count of ≥ 85,000/μL, hemoglobin ≥ 9 g/dL
10. Serum albumin ≥ 2.5 mg/dL without intravenous supplementation
11. Adequate liver function: Bilirubin <2.5 x ULN for all, AST and ALT < 3 x ULN except for patients with significant tumor burden in their liver where AST and ALT < 5x ULN is acceptable in the absence of other etiologies for transaminitis.
12. Adequate renal function: creatinine clearance [Estimating glomerular filtration rate (EGFR) method or measured] ≥ 50 mL/min. Measured clearance will be used if both numbers are available.
13. Must have left ventricular ejection fraction ≥ 50%
14. Must have an ambulatory oxygen saturation of > 88% on room air.
15. The expansion phase patients must meet all eligibility criteria above (from 1 to 14) AND must have diagnosis of pancreatic adenocarcinoma or extrahepatic cholangiocarcinoma with pathology confirmed to be consistent with one of these diagnoses by NCI Laboratory of Pathology.
16. The effects of LMB-100 alone or in combination with tofacitinib on the developing human fetus are unknown. For this reason, women of child-bearing potential and men must agree to use adequate contraception (hormonal or barrier method of birth control; abstinence) prior to study entry until 3 months the last dose of study therapy. Should a woman become pregnant or suspect she is pregnant while she or her partner is participating in this study, she should inform her treating physician immediately.
17. Ability of participant to understand and the willingness to sign a written informed consent document.

Exclusion criteria:

1. Known or clinically suspected CNS primary tumors or metastases including leptomeningeal metastases as CNS penetration of LMB-100 is expected to be poor. CNS metastases are permitted if they have been previously treated, are asymptomatic, and have had no requirement for steroids or enzyme-inducing anticonvulsants in the last 14 days.
2. Evidence of significant, uncontrolled concomitant diseases which could affect compliance with the protocol or interpretation of results, including significant pulmonary disease other than that related to the primary cancer, uncontrolled diabetes mellitus, and/or significant cardiovascular disease (such as New York Heart Association Class III or IV cardiac disease, myocardial infarction within the last 6 months, unstable arrhythmias, unstable angina, or clinically significant pericardial effusion).
3. Any known diagnoses, metabolic dysfunction, physical examination finding, or clinical laboratory finding giving reasonable suspicion of a disease or condition (other than mesothelin [+] cancer diagnosis) that would contraindicate the use of an investigational drug, interfere with tumor measurement or lead to a life expectancy of less than 6 months as judged by the investigator.
4. Contraindication to receiving prophylactic doses of low-molecular weight heparin (LMWH) or direct oral anticoagulants (DOAC) such as current active bleeding (except for grade 1 hematuria or epistaxis), recent history of significant bleeding without subsequent effective medical or surgical intervention, known history of gastric varices, uncontrolled malignant hypertension, history of coagulopathy that confers increased risk of bleeding. Patients on concurrent treatment with anti-platelet agents such as aspirin or clopidogrel are eligible if deemed to have acceptable risk of bleeding in consultation with Hematologist. Patients already receiving prophylactic or therapeutic doses of anticoagulant (heparin-based or DOAC) for at least 4 weeks with no indication of significant bleeding while on therapy are considered NOT to have a contraindication to this therapy.
5. Inability to administer or unwillingness to comply with recommended VTE prophylaxis for the duration of study treatment.
6. Prior diagnosis of hematologic malignancy
7. Active or uncontrolled infections (including tuberculosis, HIV, HBV, or HCV) or reasonable clinical suspicion of an active infection (such as cholangitis) as tofacitinib suppresses lymphocyte signaling and will impair host response to infection.
8. Latent TB infection as identified by interferon-γ release assay (IGRA). If IGRA is indeterminate, tuberculin skin test (TST) may be used to determine status.
9. Live attenuated vaccinations within 14 days prior to treatment.
10. Use of a strong inhibitor or inducer of CYP3A4 within 14 days prior to enrollment (see Flockhart Table (https://drug-interactions.medicine.iu.edu/Clinical-Table.aspx) or similarly updated source for a list of such agents)
11. Inability to take or digest oral medication.
12. Dementia or altered mental status that would prohibit informed consent.
13. Pregnant women are excluded from this study because the effects of LMB-100 and/or tofacitinib on the developing fetus are unknown and may have the potential to cause teratogenic or abortifacient effects. Because there is an unknown but potential risk for adverse events in nursing infants secondary to treatment of the mother with LMB-100 and/or tofacitinib, breastfeeding should be discontinued if the mother is treated with either of these agents.
14. Baseline QTcF interval of > 470 ms, participants with baseline resting bradycardia < 45 beats per minute, or baseline resting tachycardia >100 beats per minute.
15. Participants with contra-indication and/or history of severe hypersensitivity reactions to any components related to LMB-100 and tofacitinib.
16. Patients who have previously received LMB-100 (and therefore have high-levels of pre- existing ADA’s to drug)

*Mouse treatment experiment and pathologic grading of serositis in mouse H&E tissue samples*

Mice (*Msln^ki/+^*) were treated with tofacitinib (25 mg/kg, 200 µL volume, twice daily by OG, days 1-6) plus LMB-100 (3.5 mg/kg IV, 200 µL volume, Days 4 and 6), oral vehicle (200 µL OG, days 1-6) plus LMB-100, or oral vehicle plus IV PBS (200 µL volume IV, Days 4 and 6). Tofacitinib citrate was purchased from SelleckChem (product S5001), dissolved in DMSO to 250 mg/mL for storage, then diluted into 0.5% methylcellulose, followed by 0.05% Tween20 and PBS just before use. LMB-100 iTox was manufactured by Roche and provided for these studies through a Collaborative Research and Development Agreement. Animals were euthanized 24 hours after last treatment. Tissue specimens were fixed in 10% neutral buffered formalin solution for 48-72 h at RT (HT501128, Sigma-Aldrich, USA), then sent to Molecular Histotechnology Laboratory (MHL) Core facility for all histologic studies. Degree of serositis in pleura and peritoneum were graded by a trained veterinary pathologist (affiliated with the MHL Core) blinded to treatment groups as follows:

- Normal (0)
- Minimal (1)- increased inflammatory cells
- Mild (2)- inflammatory cells 2-5 cells thick, necrosis present focally or in small regions
- Moderate (3)- thick carpet of inflammatory cells >5 cells thick, multifocal necrosis with small bands of serocellular coagulum
- Severe (4)- thick sercellular coagulum, multifocal, hemorrhage

*Circulating Cytokine Analysis*

Blood for serum cytokine evaluation was separated within 4 hours and stored in aliquots at -80°C until use. The samples were tested using clinically validated custom V-PLEX assay plates on an electrochemiluminescence platform, according to the manufacturer’s instructions (Meso Scale Discovery).

*Collection and processing of blood for immune cell and CEC analyses*

Peripheral blood samples were collected in Cell Preparation Tubes^TM^ with sodium citrate (BD Vacutainer CPT Tubes, BD Biosciences, San Jose, CA). Peripheral blood mononuclear cells (PBMCs) were obtained by centrifugation and viably frozen in liquid nitrogen until analysis.

*Circulating endothelial cell analysis*

A minimum of 1X105 cells were acquired for each analysis. CECs were defined as negative for the hematopoietic marker CD45 (leukocyte common antigen), positive for the endothelial markers CD31 and CD146, and negative for the progenitor marker CD133. CECs were further subgrouped into viable versus apoptotic populations. Viability was defined by the absence of 7-aminoactinomycin D staining, and analysis was restricted to nucleated cells by gating on Hoechst 33342–positive cells. Data were analyzed using FlowJo Software version 10.6.1. (FlowJo LLC).

*Immune cell subset analysis*

Cells were incubated with Fc receptor blocking agent (Miltenyi Biotec) and stained for 20–30 min at 4°C with monoclonal antibodies. For analysis of Foxp3 and Ki67 expression, cells were fixed and permeabilized using a Fix/Perm buffer (eBioscience) according to the manufacturer’s instructions, then stained with anti-Foxp3 or anti-Ki67 antibody. Live cells were discriminated by means of LIVE/DEAD Fixable Aqua Dead Cell Stain (Life Technologies) and dead cells were excluded from all analyses. All flow cytometric analyses were performed using a MACSQuant Analyzer (Miltenyi Biotec). Flow cytometric data were quantified either as the median fluorescence intensity or as a percentage of cells, as indicated. Data were analyzed using FlowJo software version 10.6.1. (FlowJo, LLC). The following immunophenotypic markers were used to define immune subsets:

- CD4+ T cells ; CD8-CD4+
- CD8+ T cells ; CD8+CD4+
- Tregs ; CD8-CD4+CD25+Foxp3+
- eTregs ; CD8-CD4+CD45RA-Foxp3high
- nTregs ; CD8-CD4+CD45RA+Foxp3dim
- Naïve T cells ; CD45RA+CCR7+ CD28+ CD27+
- Effector T cells ; CD45RA+CCR7– CD28– CD27–
- EM1 T cells ; CD45RA–CCR7– CD28+ CD27+ CD3+ (CD4+ or CD8+)
- EM2 T cells ; CD45RA–CCR7– CD28– CD27+ CD3+ (CD4+ or CD8+)
- EM3 T cells ; CD45RA–CCR7– CD28– CD27- CD3+ (CD4+ or CD8+)
- EM4 T cells ; CD45RA–CCR7– CD28+ CD27- CD3+ (CD4+ or CD8+)
- CM T cells ; CD45RA–CCR7+ CD28+ CD27+ CD3+ (CD4+ or CD8+)
- PMN-MDSC ; CD14-CD11b+CD15+
- M-MDSC ; CD14+ CD11b+ HLA–DRlow/– CD15–
- eMDSC ; Lin (CD3, CD19, CD14, CD56)-HLA-DR-CD33+
- Classical monocytes ; CD14+ CD16–
- Intermediate monocytes ; CD14+ CD16+
- Non-classical monocytes ; CD14dim CD16+
- CD1c+ myeloid DC (mDC) ; lineage (CD3, CD19, CD56)−HLA-DR+CD11c+CD1c+
- CD141+ mDC ; lineage−HLA–DR+CD11c+CD141+
- CD303+ plasmacytoid DC (pDC) ; lineage−HLA–DR+CD11c+CD303+

 The following monoclonal antibodies were used:

- CD14 clone HCD14
- CD16 clone 3G8
- HLA-DR clone LN3
- CD3 clone OKT3
- CD56 clone MEM-188
- CD19 clone HIB19
- CD11b clone ICRF44
- CD15 clone W6D3
- CD33 clone WM53
- CD11c clone Bu15
- CD1c clone L161
- CD141 clone M80
- CD303 clone 201A
- CD83 clone HB15e
- CD8 clone SK1
- CD4 clone RPAT4
- CD25 clone BC96
- Foxp3 clone 206D
- PD-1 clone EH12.2H7
- CTLA-4 clone L3D10
- TIM-3 clone F38-2E2
- ICOS clone C398.4A
- GITR clone 108-17
- CD45RA clone HI100 (BioLegend)
- Ki67 clone B56 (BD Biosciences)


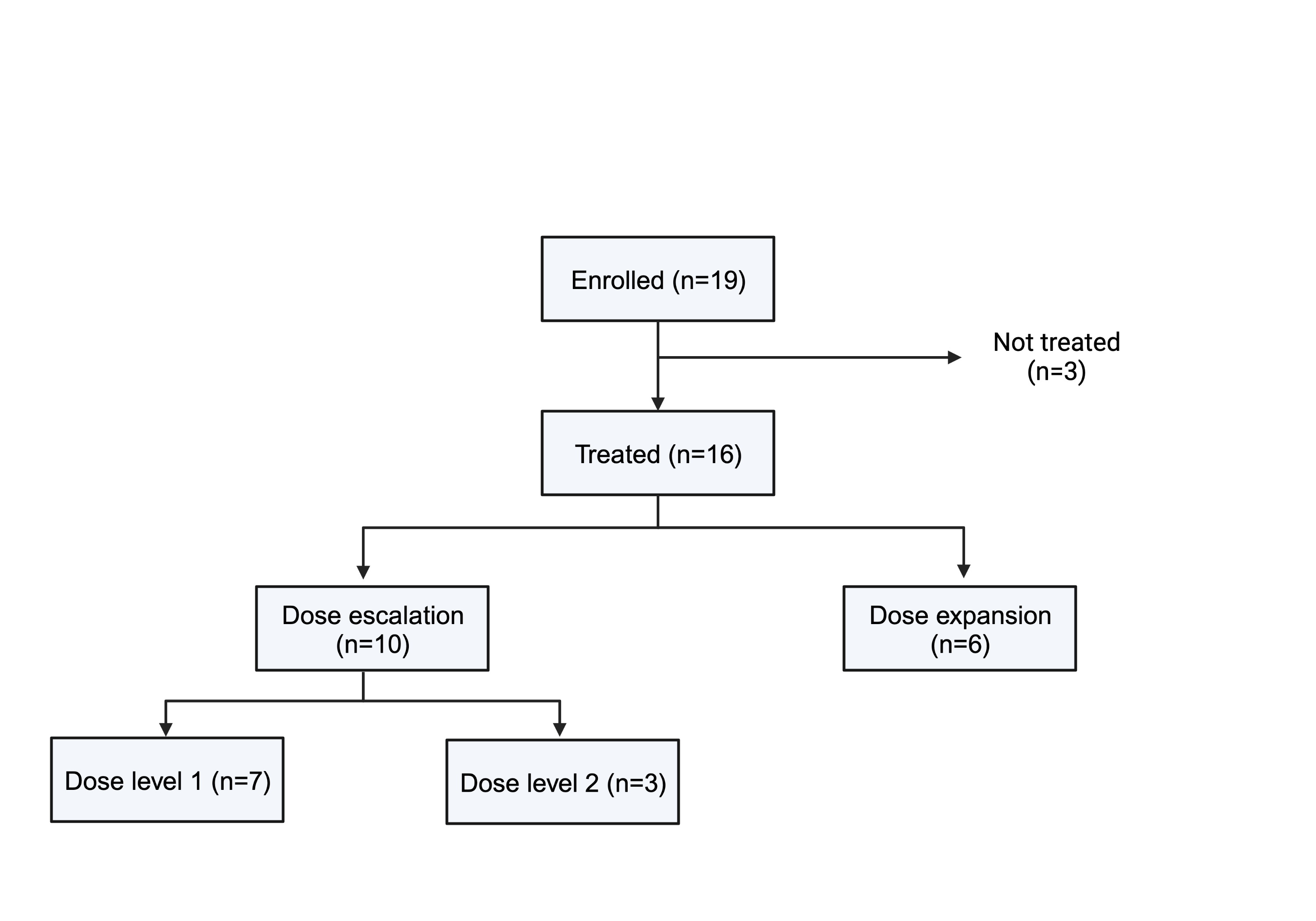


**Supplemental Figure 1**

**A**

**B**

**C**

**D**

**Supplemental Figure 1.**

A) Patient CONSORT diagram B) Summary of grade 3 or higher adverse events unrelated to study interventions. C) LMB-100 cycle 1 Cmax grouped by adverse event of cardiac inflammation. D) Percentage of peripheral blood CECs. Kruskall-Wallis non-parametric test adjusted with Dunn’s test for multiple comparisons did not identify statistically significant differences in the apoptotic CEC subset, but did identify statistically significant differences within the viable CEC subset. Levels of significance: * *p* <0.05, ** *p* <0.01

**Supplemental Figure 2**

**Supplemental Figure 2.** Best change in serum CA 19-9 as compared to baseline in evaluable patients. To be evaluable, patients must have 1) detectable CA19-9 at baseline, 2) received ≥ 1 cycle of treatment, 3) at least one re-evaluation of serum tumor marker.


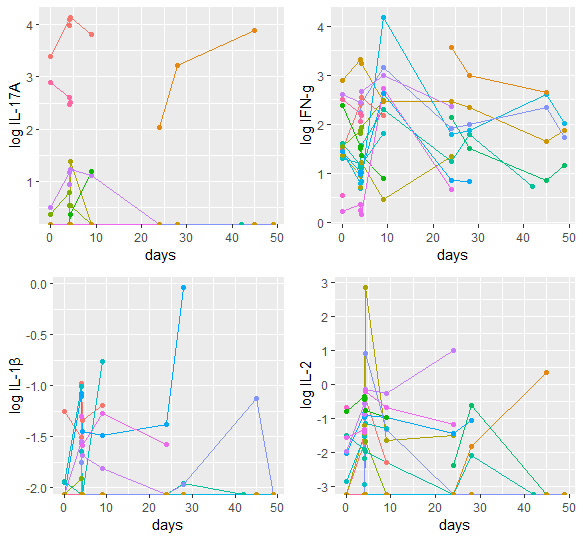

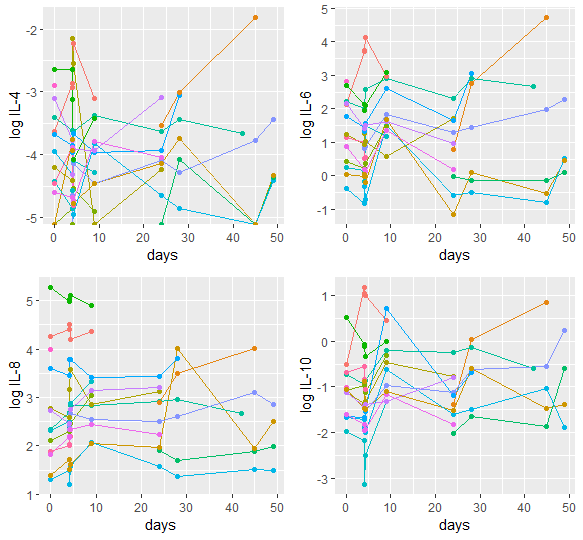

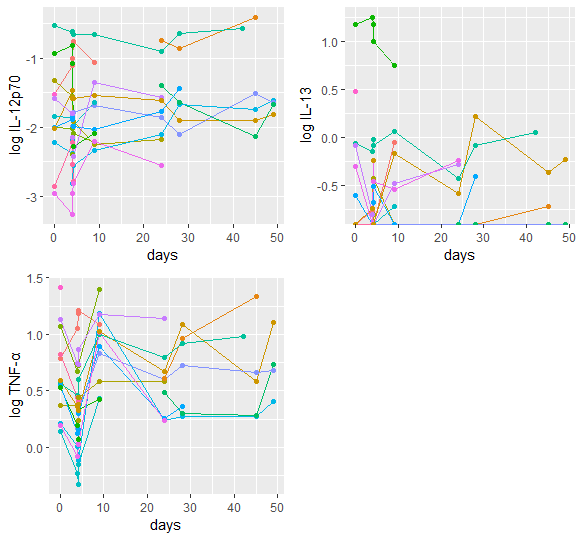


**Supplemental Figure 3**

**Supplemental Figure 3.** Peripheral cytokine concentration in individual patients. Each colored line represents one patient.

**Supplemental Figure 4.** Flow cytometric assessment of peripheral immune cells reporting intrapatient changes for all 58 cell types assessed. Each heatmap color block shows median changes in percentage of each cell subset for all patients from C1D1 until C2D4 (post-C2 tofacitinib start, but prior to LMB-100 administration), an exact Wilcoxon signed rank test was performed for each cell type, to calculate p-value. Levels of significance: * *p* <0.05, ** *p* <0.01.

**Supplemental Figure 4**


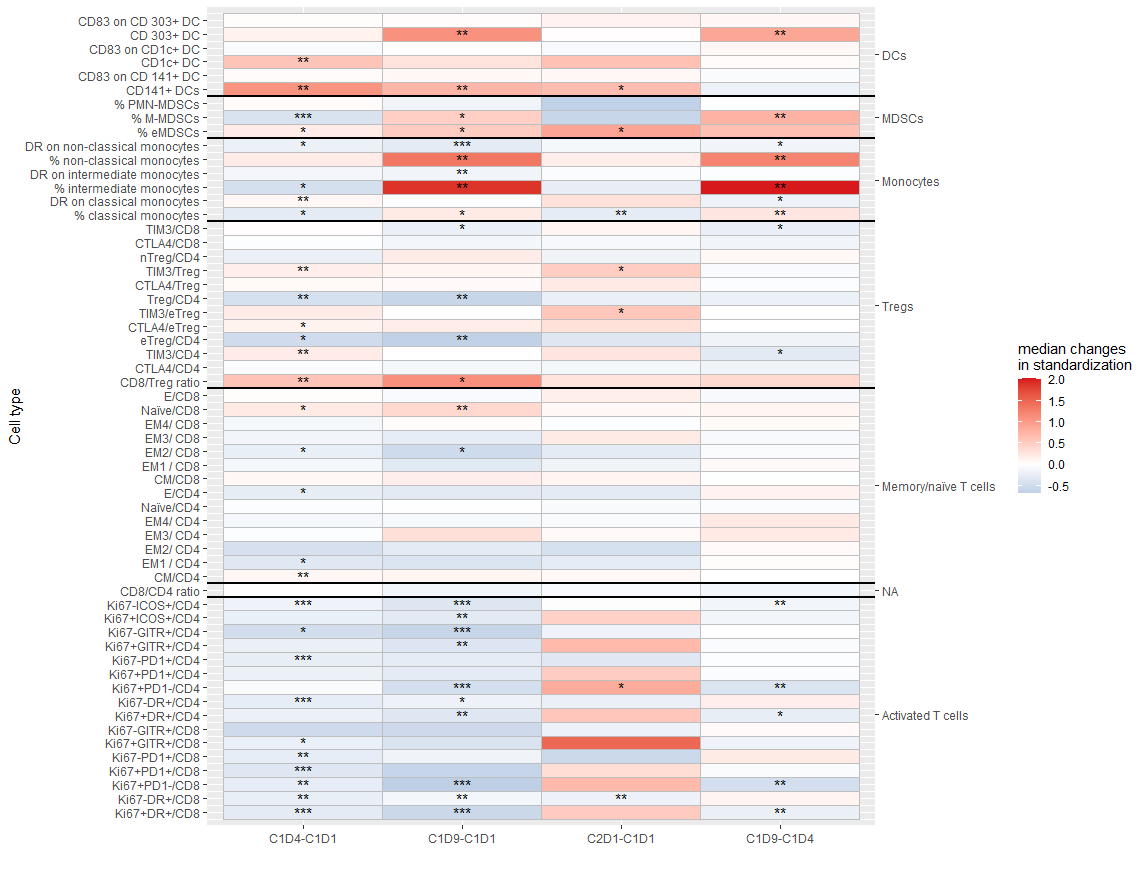

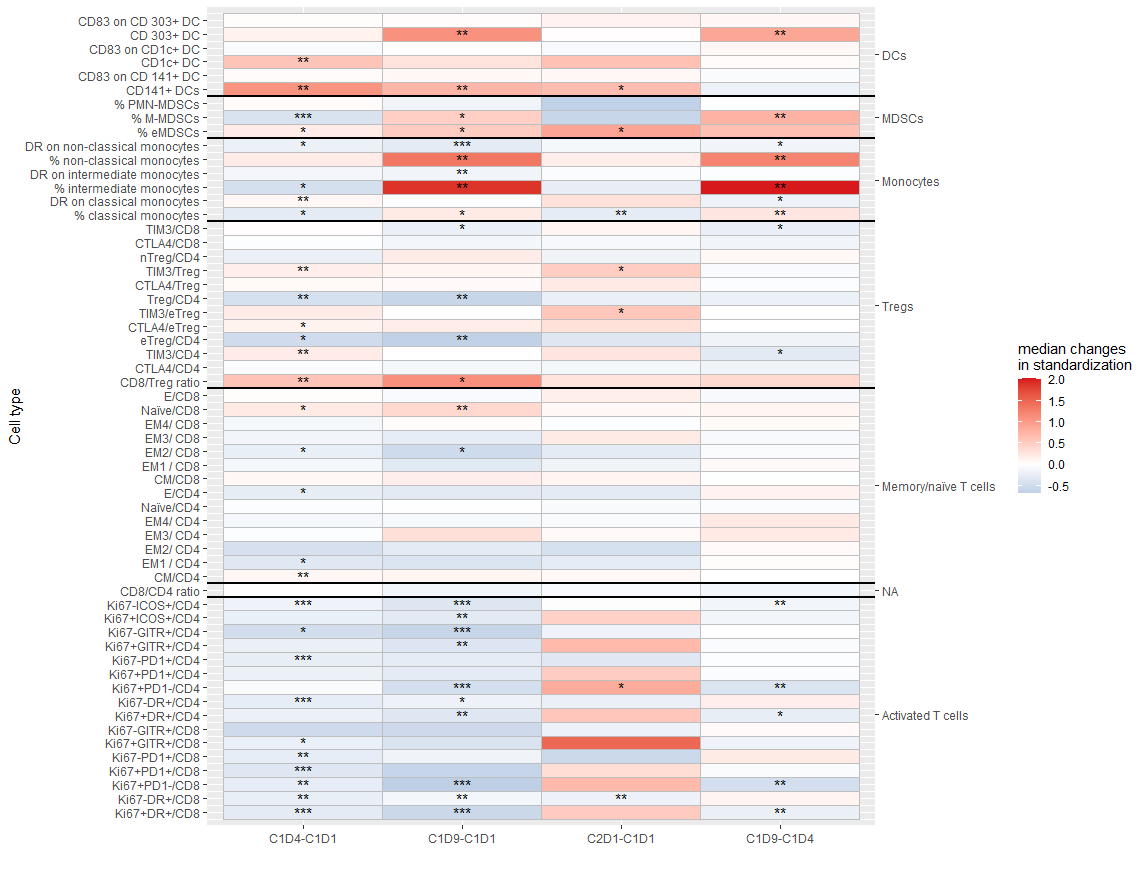

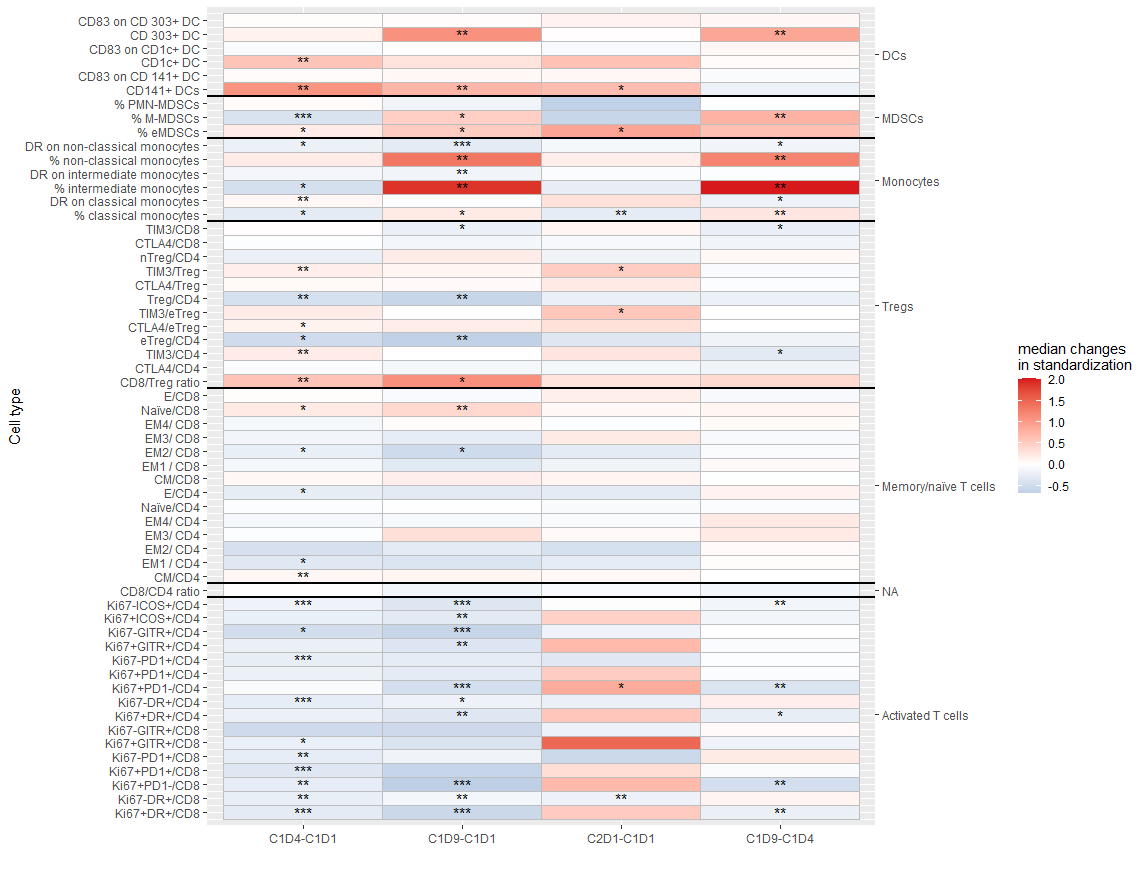


**C1D1-C2D4**

**Supplemental Table 1**

| **PK Parameter** | **100 µg/ kg (n = 12)*** | **140 µg/ kg (n = 3)*** |
| --- | --- | --- |
| Cmax (ng/mL) | 1365 (27%) | 1834 (91%) |
| AUC_LAST_ (hr*ng/mL) | 2831 (34%) | 2095 (84%) |
| T_1/2_ (hr) | 1.03 (33%) | 0.753 (29%) |
| Vd (L) | 4.5 (33%) | 5.60 (25%) |
| CL (L/hr) | 3.4 (49%) | 5.57 (52%) |

Data presented as arithmetic mean (%CV)

* 2 of 15 participants had insufficient data for calculation of above parameters (02 & 09)

**Supplemental Table 2**

|  | IL-17A | IFN-γ | IL-1β | IL-2 | IL-4 | IL-6 | IL-8 | IL-10 | IL-12p70 | IL-13 | TNF-α |
| --- | --- | --- | --- | --- | --- | --- | --- | --- | --- | --- | --- |
| baseline | 9  (69%) | 0  (0%) | 10  (77%) | 6  (46%) | 2  (15%) | 0  (0%) | 0  (0%) | 0  (0%) | 0  (0%) | 7  (54%) | 0  (0%) |
| C1D4 PRE | 9  (69%) | 0  (0%) | 7  (54%) | 5  (38%) | 0  (0%) | 0  (0%) | 0  (0%) | 0  (0%) | 0  (0%) | 8  (62%) | 0  (0%) |

**Supplemental Table 2.** Peripheral blood cytokine measurement – Summary of values below the limit of detection (lod) for each cytokine. Note: Out of 16 patients, there are 13 patients with baseline (and C1D4PRE) measurements available and the percentages are calculated based on those 13 patients. Based on these results, only seven cytokines were considered for the fold change analyses (i.e., IFN-γ, IL-4, IL-6, IL-8, IL-10, IL-12p70, and TNF- α). There are 5 (out of 61 observations) imputed values applied only for IL-4. This was done by dividing the corresponding lod value by the square root of 2.

**Supplemental Table 3**

| IL-17A |  | C1D4 EOI | C1D4 | C1D9 PRE | C2D4 PRE |
| --- | --- | --- | --- | --- | --- |
|  |  |  | 4HR POST |  |  |
|  |  | <lod ≥lod | <lod ≥lod | <lod ≥lod | <lod ≥lod |
| C1D4 PRE | <lod | 9 0 | 7 2 | 8 1 | 7 0 |
|  | ≥lod | 0 4 | 0 4 | 1 2 | 1 0 |
|  | | | | | |
| IL-1β |  | C1D4 EOI | C1D4  4HR POST | C1D9 PRE | C2D4 PRE |
| C1D4 PRE | <lod  ≥lod | <lod ≥lod  6 1  2 4 | <lod ≥lod  6 1  3 3 | <lod ≥lod  5 1  2 4 | <lod ≥lod  4 1  2 1 |
|  | | | | | |
| IL-2 |  | C1D4 EOI | C1D4  4HR POST | C1D9 PRE | C2D4 PRE |
| C1D4 PRE | <lod  ≥lod | <lod ≥lod  5 0  0 8 | <lod ≥lod  3 2  0 8 | <lod ≥lod  3 1  0 8 | <lod ≥lod  3 0  1 4 |
|  | | | | | |
| IL-13 |  | C1D4 EOI | C1D4 | C1D9 PRE | C2D4 PRE |
|  |  |  | 4HR POST |  |  |
|  |  | <lod ≥lod | <lod ≥lod | <lod ≥lod | <lod ≥lod |
| C1D4 PRE | <lod | 5 3 | 5 3 | 5 2 | 4 1 |
|  | ≥lod | 2 3 | 3 2 | 0 5 | 0 3 |

**Supplemental Table 3.** Limit of detection (lod) summary from C1D4 PRE for cytokines that were not analyzed. A contingency table for the cases where values are below lod for each time point since C1D4 PRE, and vice versa. The off-diagonal values are mostly 0 or small which implies that these changes in categorized cytokine values are not sufficient in number to be analyzed and would not provide a basis for proper statistical inference.
